# Messaging for Interventions Aiming to Improve Calcium Intake in Young Adults—A Mixed Methods Study

**DOI:** 10.3390/nu10111673

**Published:** 2018-11-05

**Authors:** Anika Rouf, Margaret Allman-Farinelli

**Affiliations:** Charles Perkin Centre, School of Life and Environmental Sciences, The University of Sydney, Sydney, NSW 2006, Australia; margaret.allman-farinelli@sydney.edu.au

**Keywords:** young adults, calcium, health promotion, social media, public health

## Abstract

Social media channels are the preferred communication tools for many young adults and therefore may have applications in health promotion. The framing of messages is important, as an intervention must resonate with the target group. The aim of this study was to determine what type of messaging is preferred by young adults to improve their calcium intake. A cross-sectional web-based survey was conducted and young adults aged 18 to 25 years recruited. A 14-item survey collected information on the participants’ demographics, ranking of text messages, mock Facebook posts with images, preferences related to type of posts they find personally relevant, and frequency and likelihood of engagement with posts and polls in social media. In addition, optional responses from participants about factors that motivate them to consume more calcium-rich foods were included and thematically analysed using NVivo. Eighty-one participants (17 males) completed the survey. No significant difference in ranking of the text messages and Facebook posts were found. Participants indicated that recipe demonstrations (*n* = 71), cost-saving tips (*n* = 70), and information on recommended daily intake (*n* = 62) were personally relevant, while meal inspiration (*n* = 70), awareness-raising posts (*n* = 41), and messages about obtaining enough calcium from non-dairy sources (*n* = 38) would encourage them to eat more calcium-rich foods. The qualitative replies indicated the tone (in young adults’ language) and length (short) of messages preferred, and the messaging they perceived would motivate young adults. In conclusion, short, aesthetically pleasing and personally relevant messages written in the language of young adults were recommended.

## 1. Introduction

Improvements in population nutrition have a pivotal role in the prevention of chronic disease. Despite large investments in preventative health campaigns [1], the diet of Australians remains a significant factor influencing non-communicable diseases [2]. Among the chronic diseases that have a nutritional component in their aetiology is osteoporosis, and attainment of peak bone mass during adolescence to young adulthood may be important for prevention [3,4]. Australian young adults are in need of an intervention to improve their currently sub-optimal calcium intakes [5]. However, young adults are a difficult-to-reach population with relatively low healthcare utilisation, which makes it difficult to disseminate information in this age group [6]. The Internet, social media, and smartphones are preferred communication and information channels for young adults [7]. The Internet has become a very popular medium for searching of health information [8,9]. However, large concerns remain about the credibility and reliability of information available online [10,11]. While young adults may be exposed to many types of health messages online, research shows they lack self-efficacy when it comes to practising these healthy dietary behaviours [12]. Interventions that aim to change an individual’s dietary behaviour should consider message framing to enhance the likelihood of uptake and resonance with the target population [13]. Previous research indicates that the way health messages are framed can affect the way it is perceived, which can vary depending on age of the message recipient [14,15]. For an intervention to be effective in changing behaviour, the messages should be motivating and be personally relevant to the individuals [16]. This idea is supported by the Elaboration Likelihood Model [17] which suggests that the effectiveness of an intervention can be enhanced by maximising personal relevance [18]. The frequency at which messages are delivered may also be important, as messages sent too often may result in loss of attention, and those sent too infrequently may result in a diluted impact. It is also important to include components that promote engagement with an intervention [19,20]. Thus, it is necessary to develop messaging that meets the needs of targeted individuals to increase motivation. The aim of this study was to conduct an online survey to identify messaging types preferred by young adults to improve their calcium intakes. 

## 2. Materials and Methods

### 2.1. Study Design 

A cross-sectional web-based survey was conducted to capture views of young adults aged 18 to 25 years. Materials and methods of the online survey were approved by the Human Research Ethics Committee at the University (HREC). The ethics approval number is 2018/079. 

### 2.2. Survey Development

The 14-item survey included basic demographic information (age, gender, whether currently studying or working), and asked for scoring of five text messages written in different tones [21]. These included an authoritative tone, with a nutrition expert to explain with scientific reasoning why a nutrition behaviour should be performed; empathetic, which explains you understand their difficulties and want to help; generation Y, that appeals to this generation and acts as a peer providing insights; solution-based, that provides tips and ideas for change; and substitution-based, that suggests how to swap one nutrition behaviour for another [21]. Five mock Facebook posts (with images) concerning breakfast were also shown to participants. All messages concerned the breakfast meal and encouraged intake of calcium-rich foods. This was created based on evidence illustrating the importance of breakfast for adequate calcium consumption [22] and our analyses of the most recent National Nutrition Survey showing the highest intakes of calcium at this meal [5]. Texts and posts were ranked by participants from 1 to 5, where a lower score indicates a greater motivator.

Additional questions were provided on the different types of information that might be personally relevant to change their eating habits, and were related to the relevance of educational posts on serving sizes and calcium requirements, health benefits, recipes and cost-saving tips for their food budget, and additional web-based resources. They were also asked about factors motivating them to eat more calcium-rich foods; whether they preferred images on every post; their views on the preferred frequency of new posts; the likelihood of them sharing Facebook posts (scale 1 to 5); and whether they would engage in on-line polls and competitions in an intervention.

Finally, participants were provided with the option to share any other comments or ideas they may have using free text. However, this was optional while all other questions were compulsory. The survey was offered online using REDCap (Vanderbilt University, TN, USA) [23] for a period of four months (March to June 2018).

### 2.3. Recruitment

**Figure 1:**
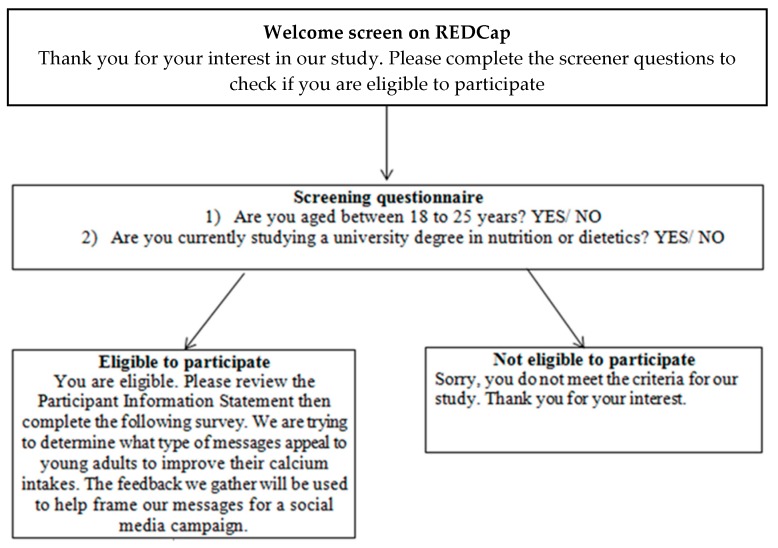
Survey recruitment process.

Young adults aged 18 to 25 years who had not completed or were not currently undertaking a nutrition-related degree were eligible to participate. Modes of recruitment included distributing flyers at university, face-to-face and over social media. Participants were provided with a website link to REDCap to complete the survey (Figure 1). The website link opened to screener questions where participants were able to check their eligibility to participate in the study. If participants were eligible, they were provided with the Participant Information Statement and a brief description of the study. On the next screen, participants were asked to provide consent and proceed to completing the survey questions. As an incentive, participants were offered the chance to win one of five $20 AUD gift vouchers for a department store. 

### 2.4. Data Analyses.

Responses from surveys were tabulated and mean scores for ranking calculated and analysis of variance (ANOVA) conducted to assess any differences in popularity of posts was performed using SPSS for Windows 22.0 software (IBM Corp. Released 20133 IBM SPSS Statistics for Windows, version 22.0 Armonk, NY: IBM Corp). The open-ended questions were analysed qualitatively using NVivo 11 (2015, version 11.0.0317, QSR International Pty Ltd., Melbourne, Victoria, Australia). Descriptive coding was selected to summarise the comments made by respondents. This approach [24] involved a thorough reading of all comments to become familiar with the data. The next step was to generate initial codes to capture the main ideas. The themes were then reviewed, finalized, and named. Finally, representative quotes [25] were organized under each of these themes to accurately depict the voice of participants and ensure that the concepts remain close to participants’ own words or terms. 

## 3. Results 

### 3.1. Sample Characteristics 

The survey was attempted by 116 participants and completed by 81 respondents. Only the responses from participants who completed the survey were included in the analyses. The mean age of the participants was 21.91 ± 2.34 years (Table 1). The majority of the respondents were female (*n* = 64) and studying full-time (*n* = 57). A smaller proportion of the participants were working full-time (*n* = 16), studying part-time (*n* = 4), or other (*n* = 4). Category “other” included recent graduates looking for work and those currently working with plans to begin studies. 

### 3.2. Quantitative Analyses

Table 2 shows the mean ranking scores for the five different text messages. Table 3 shows mock Facebook posts with images where participants were asked to rank their preference in a similar manner. The mean scores for texts ranged from 2.7 to 3.3 and for the posts ranged from 2.6 to 3.2. No significant differences in scores were found (F = 2.152, *p* = 0.074 for texts; F = 1.904, *p* = 0.109 for posts). Direct comparisons of the text messages by tone is not possible because of small differences in content and images were varied in the Facebook posts.

### 3.3. Personal Relevance 

Respondents were allowed to choose more than one answer when indicating their personal relevance. Young adults chose recipe demonstrations and meal ideas as the most preferred types of posts (*n* = 71), closely followed by cost- or money-saving tips (*n* = 70). This was followed by a post on recommended daily intake as they were not sure how much calcium they needed to consume every day (*n* = 62), and an educational post on serving size (*n* = 56). Posts regarding the health benefits of calcium and those that provided links to additional resources (i.e., current research, the dietary guidelines and other credible sources of information) were of less relevance to the respondents (*n* = 38; *n* = 45 respectively). When asked what would motivate them to eat more calcium foods, participants once again indicated that meal inspiration posts including recipe ideas and pictures (*n* = 70) would be most helpful. Awareness raising stating health benefits and the risks of not consuming enough (*n* = 41) or posts educating on non-dairy or vegan sources of calcium were less popular (*n* = 41; *n* = 38 respectively). Almost all participants indicated their preference for seeing an image with every post (*n* = 76). 

In terms of frequency of posts, the most common response was a few times a week (*n* = 33); but 10 participants said daily. Using a Likert scale, the majority of participants indicated their reluctance to tag, comment and share posts about calcium with their own Facebook friends (*n* = 52). The average score was 2.15 ± 1.0, with 1 ranked as “not likely” and 5 ranked as “highly likely”. In regard to engaging in on-line activities, over half were happy to use a voting poll (*n* = 46). A majority of participants expressed reluctance to share their food-related photo on a public social media page. 

### 3.4. Thematic Analysis of Open Comments

In total, 22 participants responded to the optional free text box at the end of the survey. The majority of the respondents were females (*n* = 19) and were studying full-time (*n* = 17). All responses from participants were thematically analysed which broadly related to their preferences for an intervention, as well as motivators to consume calcium-rich foods. 

#### 3.4.1. Tone and Text

Several participants suggested posts should be kept short and sharp at the same time, “*Short and sweet social media posts with good pictures reel me in every time*”; “*I tend to click for the recipe based on whether it looks delicious and if the ingredients seem healthy*”. One participant indicated that some of the sample posts “*were too long and I struggled to get to the end of it*”. The importance of aesthetics was highlighted by multiple participants as “*good quality photos really make it or break it for me*”. One participant indicated using pictures and serving sizes to convey the message, “*posts about food-swaps where a common food item can easily be swapped for a higher calcium version*”.

A few participants indicated that paternalistic messages should be avoided, “*I think people might disengage a bit if they see a message as paternalistic…”*. Participants suggested that hashtags should be used with caution, as illustrated by “*the hyperactive peppy hashtag thing is kind of patronising; I really haven’t heard or seen anyone of any age group talk like that*”. It was suggested that posts should be phrased appropriately, as illustrated by “*I think the most important way to get young people’s attention is to hire young people to write the posts—they know how to talk to our generation over the Internet the best, e.g., in a way that doesn’t sound condescending or boring”.* It was also proposed that the messages should be made memorable, “*I think the best way to advertise is to make an ad funny and memorable. Maybe with a catchy slogan or something that people will never forget*”.

#### 3.4.2. Personal Relevance 

In terms of personal preferences for text, participants expressed their desire to make any health messages relevant to the age group. Participants acknowledged the pressure young adults face while juggling work and study commitments and highlighted the need for including convenience options for their busy lifestyle, “*It is important to tell young adults how to consume calcium rich foods in a busy lifestyle*”. Suggested options included “*on-the-go snacks that look appealing and foods that do not show all the health benefits on face (i.e., salads)*”. 

Some participants highlighted not being able to see calcium intake as a priority, as illustrated by “*I reckon just giving people an easy avenue into increasing calcium intake and a reason to care (immediate benefits like being more sharp and strong as well as the long-term ones) will do the trick*”. It was also suggested that health messages must be made relevant to the age group, as “*stuff happening decades from now is bad but elicits less of an urgency and personal response than stuff happening in our near future or that directly impacts other goals (e.g., full-time employment, sporting achievements, academic success etc.)*”.

Participants acknowledged the challenges that young adults face which makes it difficult to put diet as a priority, as illustrated by, “*they aren’t stupid. If they’re not getting enough calcium, it probably just skipped their minds because most people are really busy juggling family and friends and life in general, not because they think it’s uncool*”. 

#### 3.4.3. Motivators 

The most commonly reported motivator was found to be taste, “*I tend to click for the recipe based on whether it looks delicious and if the ingredients seem healthy*”. A few participants highlighted the importance of cost as a motivator. One participant explained that money saving tips could act as a motivator, “*as a uni student, I find that anything to do with money saving really gets my attention*”. It was indicated that the behaviour change should seem attainable, as illustrated by, “*I think above all, getting the proper calcium intake needs to seem easy to achieve. I already know that I don’t eat well but changing my habits requires changing my mindset*”. 

Two participants suggested using ‘freebies’ and providing more examples of calcium-rich foods to get the attention of young adults, “*I think you guys should give more examples about easy get food with abundant calcium, especially, with good taste will be better, or provide some menu, or freebie on the street to us*”. Further suggestions for motivation included having more social media posts on the risks of inadequate calcium intake, as demonstrated by, “*increasing understanding of how having an adequate intake of calcium can affect more than just bone healthy; increasing understanding of all the benefits of calcium and more information on calcium sources*”. It was indicated that the negative effects of low consumption should be communicated clearly, as shown by “*makes posts that explain the detrimental health effects of not taking in calcium and at the end provide an easy nutritional recipe that can help boost it*”.

## 4. Discussion 

To our knowledge, this is the first study to examine aspects of messaging for improving calcium intake in young adults. All text messages and messages with posts scored similarly. Relevance to the age group is obviously needed and recipes and tips keenly sort. While young adults like the idea of social media posts, they are not keen to engage in sharing posts with other members in a research setting and would be unlikely to share the Facebook intervention posts with a friend.

A previous study measuring public engagement of the National Cancer Institute Facebook page reported that posts with photos received significantly more likes, comments and shares [26]. Visual content on social media can grab the attention of readers and portray information more efficiently than plain text messages [27]. For example, photos on Facebook will typically generate 53% more likes compared to a post containing text only [28]. A few respondents commented on picture quality as the most important factor, which is comparable to an online survey conducted worldwide where production quality was rated as the number one factor [29]. Moreover, the preference for meal inspiration posts and recipe demonstrations indicate the great potential of video technology in educating young adults. Mayer’s cognitive theory of digital learning explains that viewers can only process a limited amount of information at one time as visual and auditory information are processed via different pathways [30]. However, as video technology comes with pause and rewind options, it allows the learners to process more information [31]. A previous study investigating vegetable intake in Australian young adults reports similar findings, where food pictures and recipe ideas were ranked as highly preferred while awareness raising posts received a lower ranking [32]. 

To make the messaging relevant to young adults, we need to understand that they lead busy lifestyles and want suggestions for eating more calcium-rich foods to provide quick and easy options without the need to invest large amounts of time and energy into cooking [33]. The messages must also contain something of relevance for the present time in their lives rather than the threat of something in the distant future [34]. The scoring of the text messages did not differ by the tone; however, direct comparisons are not possible because of confounding by content and length. Previous research has found a preference for GenY or authoritative tone [21,32]. Our qualitative research here did indicate that the language of young adults was preferred. Gender-specific messaging may be particularly helpful [35,36]; as research suggests that young males are motivated by messages relating to physique [32,37,38]. In contrast, young females are motivated by messages that improve their self-confidence [39,40]. There was acceptance of messaging about health consequences, raising awareness and increasing knowledge, and explaining why it is important to take action, which is consistent with literature [41].

Regarding the types of messages that motivate participants to engage and change behaviour, the appeal, taste and cost of suggested foods and recipes is important. Our comments from participants showed that taste was an important factor influencing food choice which is similar to other studies conducted with young adults [42,43]. Some participants indicated that cost influenced their food choices, which is also consistent with earlier qualitative work with young people [21,43,44,45,46]. The majority of respondents were reluctant to share personal content with strangers, as has been reported previously in an Australian study involving healthy young adults [32]. Similarly, studies conducted with young adults with diabetes or common mental health disorders reveal that many choose not to disclose health experiences to avoid potential negative implications for identity construction [47]. In particular, Facebook has been identified as a space for constructing personal and group identity [48,49,50,51] which means that posting or “liking” content can mirror offline social interactions and be seen as performing to identity [52]. The users are concerned about how audiences might view their disclosure and even the seemingly innocuous act of “liking” pages can affect their management of identity [47].

## 5. Study Limitations and Future Implications 

The strengths of this study include the large sample size which has allowed for the generation of quantitative and qualitative data. While most of our respondents included 18 to 25-year-olds enrolled in tertiary education, some were also employed in the workforce. A limitation of the study was the underrepresentation of young males, as they were far from reaching equal proportions. However, this is similar to other Australian research that has found it more difficult to recruit men into population studies [53]. To reduce the burden on the respondents, only one set of messages and one set of Facebook posts were tested. No conclusions about the preferred tone of messages can be made because of confounding by length of messages as the qualitative research revealed length matters and variations in content is another confounder; using only one set of messages limits the generalizability of the scoring; the posts made with the images are not directly comparable because different foods were depicted each time and this confounds rankings. The qualitative comments provided insights on the preferred tone and type of messages and showed that images are well received. The findings of this research were part of formative evaluation for an intervention aimed to improve calcium intake in Australian young adults. Therefore, caution should be exercised when interpreting these findings for other groups, especially those from other countries or with different cultures. 

## 6. Conclusions

Personal relevance of messages and targeting motivations, as well as tone, length and aesthetics must be considered in developing social media based interventions. The findings captured in this survey together with findings of previous research will be used to refine the design of a Facebook intervention to improve calcium intake in young adults. 

## Figures and Tables

**Table 1:** Survey participant characteristics (*n* = 81).

Characteristics	*n*
Males	17
Age, years	
18–20	21
21–25	60
Occupation	
Full-time student	57
Part-time student	4
Working full-time	16
Other	4

**Table 2:** Participants rating of messages as the most motivating to increase calcium-rich food and the mean score (1 is the highest motivation and 5 is the lowest motivation possible).

Message	Tone of Voice	Mean Score
It is important to get your calcium every day as it can lower your risk of chronic diseases. If you are not meeting your recommendations, it is time to make to a change and start being a healthier you at breakfast!	Authoritative	3.3
We understand that it is hard for you to eat healthy when you have a very busy life, but planning your meals early will make it easier. You could start with planning your breakfast the night before with something as simple as yoghurt and muesli?	Empathetic	3.1
Plan your brekkie the night before and step up your brekkie game! Add some milk or yoghurt and you’re good to go #brekkielikeaboss	Generation Y	2.8
Did you know that breakfast is an easy way to boost your calcium intake? Start your day by pouring some milk on your cereal or top some yoghurt on your muesli.	Solution-based	2.7
Being in a rush in the morning can often mean grabbing breakfast from outside. Why not start planning your breakfast the night before to save yourself from making unplanned purchases in the morning?	Substitution-based	3.0

**Table 3:** Number of participants rating Mock Facebook post as the most motivating to increase calcium-rich foods and the mean score (1 is the highest motivation and 5 is the lowest motivation possible).

Mock Post Example	Mean Score
Research has shown that consuming breakfast is associated with positive health outcomes including improved cognitive function and memory. If you are not a breakfast eater, it is time to make a change and look after yourself! Why don’t you start with this overnight chia pudding? Like this post if you want us to share the recipe. 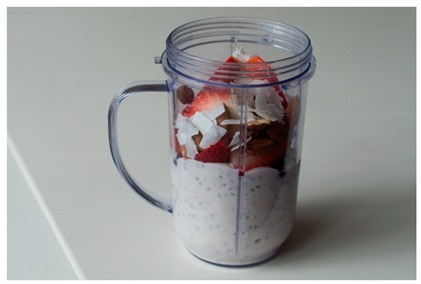	3.1
We know that it is difficult to wake up early for breakfast because sleep is so precious! And this is why we have created easy granola recipes like these so you can get the best of both worlds. Like this post if you want us to share the recipe. 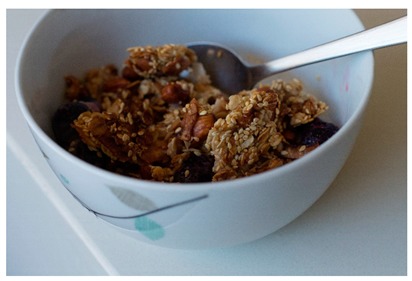	3.2
Need a sweet fix? We’ve got you covered with this delicious chocolate mousse. We have used ricotta and almonds here to bump up the calcium content. Like this post if you want us to share the recipe. 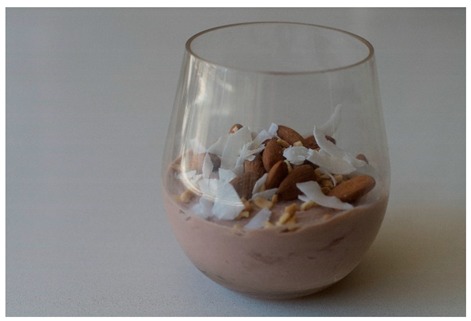	2.6
Craving a sweet dessert? Satisfy your craving with these cute pikelets that are calcium-rich and delicious. Like this post if you want us to share the recipe. 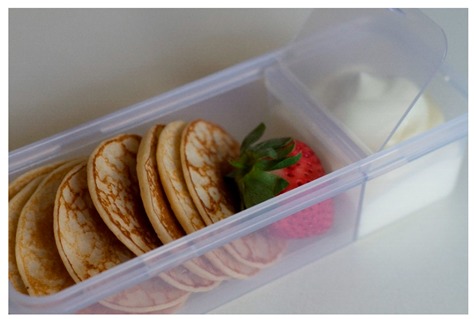	3.1
Instead of purchasing pancakes from outside, why don’t you make some on your own? These calcium-rich ricotta pancakes will curb your sweet craving and keep you feeling satisfied! Like this post if you want us to share the recipe. 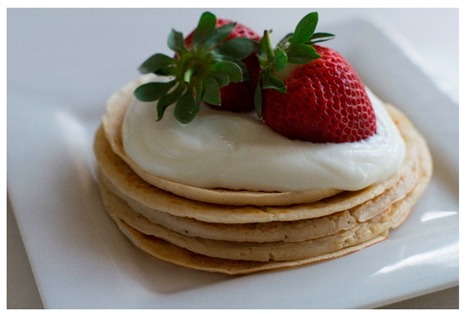	3.0
